# Pulmonary vein and left atrial posterior wall isolation using a balloon-in-basket pulsed-field ablation catheter via the superior vena cava approach in a patient with interrupted inferior vena cava: a case report

**DOI:** 10.1093/ehjcr/ytag345

**Published:** 2026-05-11

**Authors:** Kohei Ukita, Hannes Alessandrini, Charlotte Eitel, Roland Richard Tilz

**Affiliations:** Department of Rhythmology, University Heart Center, University Hospital Schleswig-Holstein, Ratzeburger Allee 160, Lübeck 23562, Germany; Department of Rhythmology, University Heart Center, University Hospital Schleswig-Holstein, Ratzeburger Allee 160, Lübeck 23562, Germany; Department of Rhythmology, University Heart Center, University Hospital Schleswig-Holstein, Ratzeburger Allee 160, Lübeck 23562, Germany; Department of Rhythmology, University Heart Center, University Hospital Schleswig-Holstein, Ratzeburger Allee 160, Lübeck 23562, Germany

**Keywords:** Atrial fibrillation, Case report, Catheter ablation, Pulsed field ablation, Pulmonary vein isolation, Superior vena cava

## Abstract

**Background:**

Pulmonary vein isolation (PVI) is the cornerstone of catheter ablation for atrial fibrillation (AF) and is typically performed via transfemoral access. In patients with interrupted inferior vena cava (IVC), however, alternative access routes such as the superior vena cava (SVC) approach are required. A balloon-in-basket (BiB)-pulsed field ablation (PFA) is a novel non-thermal ablation modality that offers stable positioning and uniform tissue contact.

**Case summary:**

A 66-year-old man with symptomatic persistent AF, characterized by palpitations and interrupted, IVC was referred to our institution. Catheter ablation using a BiB-PFA system via the SVC approach was planned. Transseptal puncture was successfully achieved under transesophageal echocardiographic and fluoroscopic guidance. PVI was achieved using the BiB-PFA catheter. Additional left atrial posterior wall (LAPW) isolation was performed with selective electrode activation to avoid anterior wall delivery. The procedure was completed without complications. At the 3-month follow-up, no recurrence of atrial tachyarrhythmia was detected on Holter monitoring, and the patient remained asymptomatic.

**Discussion:**

This case highlights the feasibility and safety of performing PVI and LAPW isolation via the SVC approach using the BiB-PFA system in a patient with interrupted IVC. The BiB-PFA system provides stable catheter manipulation and allows targeted lesion delivery through selective electrode activation. Although limited by its single-case nature and short follow-up, this report suggests that the BiB-PFA system may expand the therapeutic options for patients with complex venous anatomy.

Learning points

- In rare cases of interrupted inferior vena cava (IVC), alternative access routes such as superior vena cava (SVC) approach are required for catheter ablation.
- The balloon-in-basket (BiB)-pulsed field ablation (PFA) system enables stable positioning, uniform tissue contact, and selective electrode activation via SVC approach.
- Catheter ablation using the BiB-PFA system via SVC approach is feasible and may broaden therapeutic options for patients with challenging venous anatomy.

## Introduction

Pulmonary vein isolation (PVI) is the cornerstone of catheter ablation for atrial fibrillation (AF) and is typically performed via a femoral vein approach.^1^ In rare cases of interrupted inferior vena cava (IVC), however, alternative access such as a superior vena cava (SVC) approach is required.^2^

Pulsed field ablation (PFA) has recently emerged as a non-thermal modality based on irreversible electroporation, allowing myocardial tissue selectivity while minimizing the risk of collateral damage.^3^ Among available PFA systems, the balloon-in-basket (BiB)-PFA catheter, an innovative device combining a compliant balloon with an expandable multielectrode lattice structure, provides stable catheter positioning and consistent electrode-tissue contact.^4^

In this report, we describe a case of PVI and additional left atrial posterior wall (LAPW) isolation using a BiB-PFA catheter via the SVC approach.

## Summary figure

**Figure:**
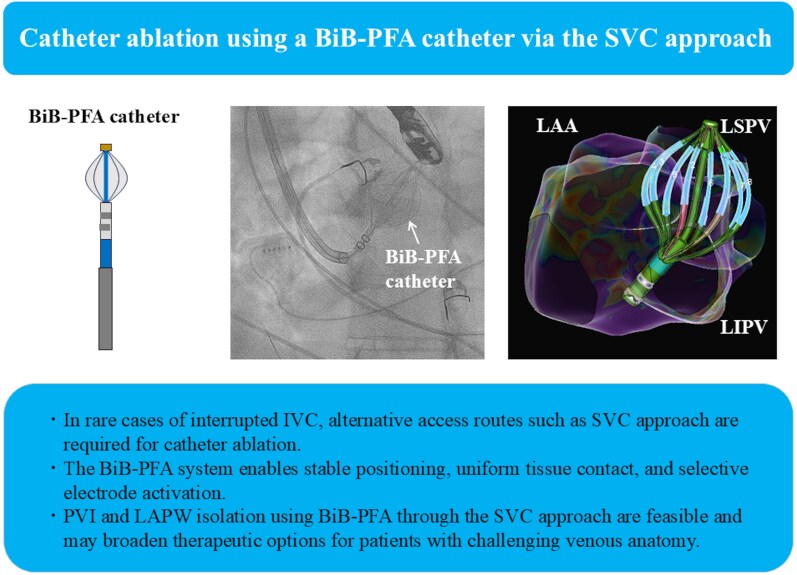


## Case report

A 66-year-old man with symptomatic persistent AF presenting with frequent palpitations had previously undergone an attempted catheter ablation at a referring hospital 3 months earlier. The procedure was discontinued due to the interrupted IVC, and the patient was referred to our institution for further management. The patient had no significant comorbidities. Thromboembolic risk assessment revealed a CHA_2_DS_2_-VASc score of 1, and bleeding risk assessment showed a HAS-BLED score of 1. He had no antiarrhythmic drugs prior to referral and was receiving apixaban (10 mg/day). Physical examination revealed an irregular heartbeat. A 12-lead electrocardiogram revealed AF with a heart rate of 127 beats per minute (*Figure 1A*). Laboratory tests showed an elevated NT-proBNP level of 250 pg/mL. Echocardiography showed left atrial enlargement (left atrial diameter: 52 mm) and normal left ventricular systolic function. Catheter ablation using the BiB-PFA system via the SVC approach was planned.

**Figure 1:**
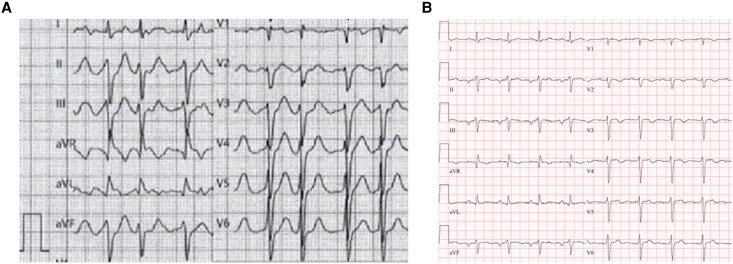
Pre- and post-procedural 12-lead electrocardiograms. (*A*) 12-lead electrocardiogram before the ablation procedure showing atrial fibrillation (25 mm/s, 10 mm/mV). The heart rate was 127 beats per minute. (*B*) 12-lead electrocardiogram after the ablation procedure demonstrating restoration and maintenance of regular R-R interval rhythm (25 mm/s, 10 mm/mV). The heart rate was 86 beats per minute.

The ablation procedure was performed under deep sedation achieved with midazolam, fentanyl, and propofol. A transesophageal echocardiography (TEE) probe was inserted. A decapolar catheter was advanced into the coronary sinus via an ultrasound-guided puncture of the left axillary vein. Additional access was obtained via the right internal jugular vein, and a long sheath (SL1™, Abbott) was inserted. Transseptal puncture was successfully performed using a manually pre-shaped transseptal needle (BRK™, Abbott) under TEE and fluoroscopic guidance (*Figure 2A*), and intravenous heparin was administered to maintain an activated clotting time (ACT) > 300 s. The SL1 sheath was exchanged for a steerable 13 Fr sheath (Agilis™ NxT, Abbott) over a 0.035-inch stiff wire (Amplatz Super Stiff™, Boston Scientific) (*Figure 2B*). After advancing the tip of the Agilis into the left atrium (LA), a BiB-PFA catheter (Volt™, Abbott) was introduced (*Figure 2C*). The three-dimensional geometry of the LA and pulmonary veins (PVs) was reconstructed using the BiB-PFA catheter with an electroanatomical mapping system (EnSite™ X, Abbott). A voltage map was created during AF rhythm (*Figure 3A*). PFA applications were delivered to each PV at 1800 V (three applications to the left superior PV [*Figure 3B* and *C*], two to the left inferior PV, three to the right inferior PV, and two to the right superior PV). Subsequently, additional LAPW isolation was performed because low voltage areas (<0.5 mV) were identified in the LAPW on the pre-ablation voltage map.^5,6^ Since the approach differed from the usual case, it had been predetermined that additional LAPW isolation would be performed if low voltage areas were identified in the LAPW. Two applications were delivered to the LAPW at 1800V, selecting electrodes facing the posterior wall and deselecting those facing the anterior wall (*Figure 3D* and *E*). Sinus rhythm was restored during this application. A postablation voltage map was created during sinus rhythm (*Figure 3F*). Phrenic nerve capture was verified with SVC pacing.

**Figure 2:**
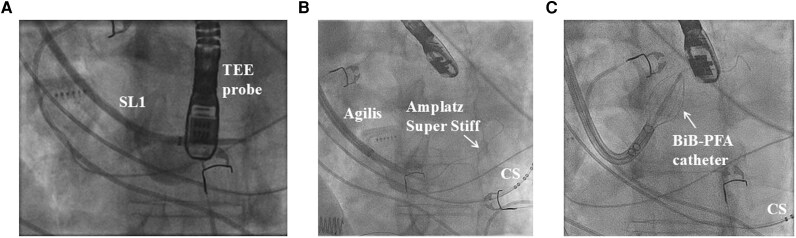
Fluoroscopic images illustrating the superior vena cava approach during the ablation procedure. (*A*) Transseptal access was obtained with SL1™ (Abbott) (30-degree left anterior oblique view). (*B*) The SL1 sheath was exchanged for Agilis™ NxT (Abbott) over Amplatz Super Stiff™ (Boston Scientific) (35-degree left anterior oblique view). (*C*) The BiB-PFA catheter (Volt™, Abbott) was introduced into the left atrium (20-degree left anterior oblique view). BiB, balloon-in-basket; CS, coronary sinus; PFA, pulsed field ablation; TEE, transesophageal echocardiography.

**Figure 3:**
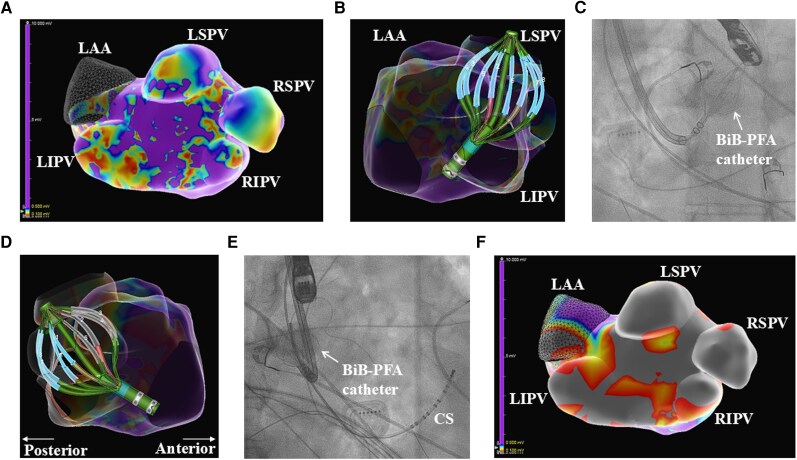
Electroanatomical mapping and pulsed-field ablation using the balloon-in-basket catheter via the superior vena cava approach. (*A*) Pre-ablation voltage map obtained with the BiB-PFA catheter during atrial fibrillation rhythm (posteroanterior view). (*B*) EnSite™ X (Abbott) image during application to the left superior pulmonary vein at 1800V (left lateral view). (*C*) Fluoroscopic image during application to the left superior pulmonary vein (20-degree left anterior oblique view). (*D*) EnSite™ X image during application to the left atrial posterior wall at 1800V (right lateral view). During this application, electrodes facing the posterior wall were selected (No. 1, 6–8), while those facing the anterior wall were deselected (No. 2–5). (*E*) Fluoroscopic image during application to the left atrial posterior wall (20-degree right anterior oblique view). (*F*) Post-ablation voltage map obtained with the BiB-PFA catheter during sinus rhythm (posteroanterior view). BiB, balloon-in-basket; CS, coronary sinus; LAA, left atrial appendage; LIPV, left inferior pulmonary vein; LSPV, left superior pulmonary vein; PFA, pulsed field ablation; RIPV, right inferior pulmonary vein; RSPV, right superior pulmonary vein.

The total procedure time from sheath insertion to removal was 118 min, with a fluoroscopy time of 39 min. During the procedure, ACT was measured every 30 min, with a mean value of 308 s. Heparin was not antagonized. Venous access sites were managed with manual compression.

No periprocedural complications occurred, including access site-related complications. The patient was monitored for 48 h due to the use of alternative venous access and was discharged on the second day of the procedure. The 12-lead electrocardiogram at discharge is shown in *Figure 1B*. Oral anticoagulation with apixaban was continued for 3 months after the procedure, and flecainide was administered only during the first 2 months by the institutional protocol. Rhythm follow-up after discharge was performed using a single Holter monitoring and a 12-lead electrocardiogram at 3 months, with no recurrence of atrial tachyarrhythmias observed (*Table 1*). Additionally, no symptoms suggestive of recurrence were noted, including during the blanking period.

**Table 1:** Timeline of events

Day 0	Catheter ablation for atrial fibrillation was performed.
Day 2	The patient was discharged without complications.
Day 90	No recurrence of atrial tachyarrhythmia was documented on Holter monitoring, and the patient remained asymptomatic.

The patient provided written informed consent for the ablation procedure and agreed to the publication of his case details and images in this report.

## Discussion

This case demonstrates the feasibility and safety of performing PVI and LAPW isolation using a novel BiB-PFA catheter via the SVC approach in a patient with interrupted IVC.

Interrupted IVC is a rare anomaly, occurring in approximately 0.15% of the general population.^7^ In such cases, conventional transfemoral access is not possible, necessitating an alternative approach for PVI. Previous reports have described the feasibility of catheter ablation for AF performed via the SVC approach using radiofrequency ablation, cryoablation, or PFA with a pentaspline catheter.^2,8–11^

Compared with radiofrequency ablation, PFA requires larger ablation catheters and delivery sheaths, potentially reducing maneuverability, particularly with the SVC approach. Nevertheless, PFA was selected for this patient following consultation, owing to its lower risk of collateral damage. Among available PFA systems, the BiB-PFA catheter offers uniform electrode distribution, improved wall contact, and precise positioning through integration with electroanatomical mapping.^4^

Moreover, the BiB-PFA system enables selective electrode activation, which is particularly valuable for LAPW isolation in anatomically challenging cases. The ability to deselect electrodes not facing the tissue enhances procedural flexibility, allowing lesion sets to be tailored to atrial anatomy and procedural goals.

Several limitations should be acknowledged. First, the diagnosis of interrupted IVC was established at the referring hospital, and an image demonstrating interrupted IVC is not available in this case. Second, conclusions regarding safety and efficacy are limited by the single-case design and short follow-up.

Further studies are needed to evaluate the reproducibility, long-term outcomes, and safety profile of this approach in larger cohorts.

## Conclusions

In this single-case experience, PVI and LAPW isolation using a BiB-PFA catheter via the SVC approach was technically feasible and performed without complications.

## Data Availability

No new data were generated or analyzed in support of this paper.

## Lead author biography

Dr. Kohei Ukita graduated from Osaka University in 2015. After completing his residency, he worked at Osaka Rosai Hospital. Since 2025, he is an Electrophysiology Fellow at the University Hospital Schleswig-Holstein.
